# Effect of Phytobiotic Composition on Production Parameters, Oxidative Stress Markers and Myokine Levels in Blood and Pectoral Muscle of Broiler Chickens

**DOI:** 10.3390/ani12192625

**Published:** 2022-09-30

**Authors:** Karolina A. Chodkowska, Paulina A. Abramowicz-Pindor, Anna Tuśnio, Kamil Gawin, Marcin Taciak, Marcin Barszcz

**Affiliations:** 1 Krzyżanowski Partners Sp. z o.o., Zakładowa 7, 26-670 Pionki, Poland; 2 AdiFeed Sp. z o.o., Opaczewska 43, 02-201 Warsaw, Poland; 3 Department of Animal Nutrition, The Kielanowski Institute of Animal Physiology and Nutrition, Polish Academy of Sciences, Instytucka 3, 05-110 Jablonna, Poland

**Keywords:** phytobiotics, blood indices, breast muscle, cytokines, oxidative stress, chickens

## Abstract

**Simple Summary:**

Intensive rearing of broiler chickens is accompanied with pathological processes occurring in muscle tissue that decrease meat quality. The application of common spices as feed additives for chickens may improve the birds’ health and prevent the development of myopathies. Therefore, the aim of the study was to examine the effect of the dietary level of a phytobiotic composition on the production parameters, oxidative stress markers and myokine levels in the blood and pectoral muscle of broiler chickens. The composition consisted of red pepper fruit, white mustard seed, soapwort root, calamus rhizome, and thymol, and it was tested at two levels, i.e., 60 and 100 mg/kg diet. The results showed that dietary supplementation with phytobiotic composition at the level of 100 mg/kg diet improved feed efficiency in broiler chickens and might improve the quality and economy of broiler meat production. The plant constituents exerted their beneficial effects on meat via decreasing tumor necrosis factor-α concentration in pectoral muscle and increasing interleukin-6 content in the blood of chickens.

**Abstract:**

The aim of this study was to evaluate the effect of dietary level of a phytobiotic composition (PBC) on production parameters, oxidative stress markers and cytokine levels in the blood and breast muscle of broiler chickens. The experiment was performed on 48 one-day-old female Ross 308 broiler chickens divided into three groups (*n* = 16) fed the control diet (without PBC), and a diet supplemented with 60 or 100 mg/kg of PBC. After 35 days of feeding, blood and breast muscle samples were collected for analyses. There was no effect on final body weight and feed intake but PBC addition (100 mg/kg) improved feed efficiency as compared to the control. Also, this dietary level of PBC contributed to an increase in interlukin-6 content in blood and a reduction in tumor necrosis factor-α concentrations in pectoral muscle in comparison with the control group. In conclusion, the addition of 100 mg/kg PBC improved the production parameters of broiler chickens and beneficially influenced the regeneration and protection of pectoral muscle against pathophysiological processes that may occur during intensive rearing.

## 1. Introduction

Since the ban on the use of antibiotic growth promoters in 2006 in the EU and in 2017 in the US, feed and pharmaceutical companies have been working on effective alternative substances. Nowadays, the manufacturers of feed additives are faced with two tasks, i.e., production of additives with a bactericidal effect and also stimulating the growth and development of good-quality muscle tissue. There are over a dozen solutions on the market that offer the above-mentioned properties at different levels of effectiveness. The efficacy of some of them has been confirmed both at the laboratory level and on farms [1,2,3,4].

A phytobiotic composition (PBC) of red pepper fruit, white mustard seed, soapwort root, calamus rhizome and thymol has been previously used as a support for coccidiosis control [5,6]. However, these ingredients are known to play a role in processes related to muscle growth, immunity, and oxidative stress [7,8,9]. Red pepper (*Capsicum annuum* L.) fruit contains capsaicin (0.4–2.1%) [10], dihydrocapsaicin and other bioactive compounds such as carotenoids, vitamins A and C, flavonoids, steroidal saponins and volatile oils [11]. Capsaicin has antioxidant, anti-inflammatory, antiallergenic, and anticarcinogenic effects [12]. Red pepper beneficially affects growth performance of poultry, which has been shown for broiler chickens [12] and Japanese quails [13]. As a feed additive it enhances the activity of pancreatic and intestinal enzymes and bile acid secretion and leads to an increase in body weight. It has also been shown to reduce heat stress and improve nutrient digestibility, feed intake, feed efficiency, carcass traits, blood parameters and production costs [12]. Hot red pepper has a broad spectrum of antibacterial activity against gram-positive and gram-negative bacteria in broiler chickens [14]. Studies on Japanese quails fed a diet supplemented with red pepper oil also demonstrated a reduction of cecal populations of pathogens, i.e., *Salmonella* spp., coliform and *Escherichia coli* [13].

White mustard (*Sinapis alba* L.) seeds are an important source of glucosinolate sinalbin [15], of which content ranges from 123–174 μmol/g dry matter depending on the cultivar [16]. Enzymatic hydrolysis of sinalbin by myrosinase releases *p*-hydroxybezyl isothiocyanate with strong antimicrobial and anti-insecticidal properties [17,18].

Soapwort (*Saponaria officinalis* L.) root is a natural source of triterpenoid saponins, of which content reaches 21% [19]. These compounds are useful constituents of phytobiotic composition because they block and damage the membrane receptors of pathogens [20] and may exert an antioxidant effect, which has been shown for Japanese quails [21].

Calamus (*Acorus calamus* L.), turmeric (*Curcuma longa* L.) and thymol may also be used as feed additives. Calamus is mainly known for its antifungal, anti-inflammatory, antitumor, antioxidant and antidiabetic properties [22] but is also used to stimulate appetite and digestion [23]. Its bioactive constituents include: essential oils, phenols, tannins, anthocyanins and resin [23,24]. Turmeric, which contains curcuminoids, stimulates digestion, improves regeneration of hepatocytes and intestinal epithelium and exerts antioxidative, anti-inflammatory and anticoccidial effects [25,26,27,28]. Thymol and its isomer—carvacrol are constituents of essential oils, which stimulate secretory and immune functions of the digestive tract. They also cause smooth muscle relaxation. Thymol can be used as an antiseptic and antiparasitic drug [29].

Previous studies have shown that selected nutrients, including herbs, may directly stimulate myokine synthesis and so indirectly affect physiological and pathological processes in various tissues [30]. Myokines are cytokines and growth factors secreted by muscles or by infiltrating inflammatory cells during muscle regeneration. These cytokines are synthesized and secreted by myocytes during muscle contraction and have been shown to control skeletal muscle homeostasis, regeneration, inflammation and growth, in part by regulating critical muscle stem cell (satellite cell) functions. Myokines are able to regulate muscle metabolism in an autocrine, as well as a paracrine and endocrine way [31]. They may also affect different organs and tissues such as the bones, brain, liver and adipose tissue. These cytokines act together in the body and may have beneficial or non-beneficial effects on the onset of various physiological disorders and their respective outcomes. There are several myokines that have been under intensive investigation in the past several years. However, most studies have been conducted on mammals and only a few have been related to birds. The three myokines that were chosen for this study, i.e., tumor necrosis factor-α (TNF-α), interleukin-6 (IL-6) and myostatin (MSTN), have previously been described as related to satellite cells, muscle cell proliferation and differentiation, myogenesis and hypertrophy, as well as anti- and proinflammatory processes [32] that may determine the subsequent weight and quality of the poultry carcass.

As there are plenty of published articles on single herbal plants containing phytocompounds affecting health and growth performance, the effect of their mixtures has not been studied enough. Only a few previous studies have presented links between selected broiler production parameters and myokine levels. This study is the first where the effect of a PBC containing red pepper fruit, white mustard seed, soapwort root, calamus rhizome and thymol has been evaluated in broilers. The aim of this study was to determine the effect of dietary level of PBC on production parameters, oxidative stress markers and myokine levels in the blood and pectoral muscle of broiler chickens.

## 2. Materials and Methods

The design of the study, animal care, and experimental procedures were approved by the animal welfare body of The Kielanowski Institute of Animal Physiology and Nutrition, Polish Academy of Sciences (Jabłonna, Poland), in accordance with the principles of the European Union and the Polish Law on Animal Protection.

### 2.1. Animals and Diets

The experiment was performed on 48 one-day-old female Ross 308 broiler chickens, purchased from a local commercial hatchery. Birds were divided into three groups (*n* = 16) fed cereal-based starter, grower and finisher diets without the addition of PBC (control) or supplemented with 60 or 100 mg/kg of PBC (AdiCox^®^ AP, AdiFeed Sp. z o.o., Warsaw, Poland). The PBC consisted of products from the processing of spices and seasonings (red pepper fruit, white mustard seed, turmeric), products from the processing of herbs (soapwort root, calamus rhizome), vegetable oil and palm oil (hydrogenated), aroma (500 mg/100 g) and iron sulphate monohydrate (690 mg Fe/100 g). The constituents are listed in order from the highest to the lowest proportion in the composition. As declared by the producer, the PBC was rich in phytoncides and phytoalexins and contained not less than 1% of phenolic acids and not less than 0.8% of essential oils. The nutrient content of this product was as follows: 88.0% dry matter, 12.6% crude protein, 17.5% crude fibre, 7.0% crude fat, 7.4% crude ash, 0.52% lysine, 0.17% methionine and 0.03% sodium.

Starter diets were given from day 1 to day 10, grower diets from day 11 to day 28 and finisher diets from day 29 until slaughter at 35 days of age. Birds had free access to feed and water throughout the trial. Formulation of diets is shown in Table 1.

For the first 7 days of life, chickens were kept in electrically heated battery brooders with a wire-mesh floor in groups of eight birds each and fed the appropriate experimental diet. During this period the room temperature was maintained at 32 °C ± 1 °C and the light-dark cycle was set at 18/6 h. At eight day of life, chickens were moved to individual cages with wire-mesh floors and feeding of the respective experimental diets was continued until slaughter. Chickens were kept under controlled conditions at a room temperature of 32 °C, which was gradually decreased according to normal management practice, and at 18/6 h light-dark cycle. Body weight and feed intake were measured weekly, and feed conversion ratio (FCR) was calculated for each period as feed to gain ratio. At 35 days of age, chickens were sacrificed by decapitation and exsanguination. Blood was collected into heparinised tubes, centrifuged (3350× *g*, 10 min, 4 °C), and plasma was stored at −80 °C until further analyses. Pectoral muscle samples were taken, snap-frozen in liquid nitrogen and stored at −80 °C. Blood and muscle tissue samples were taken from 10 birds per group.

### 2.2. Nutrient Analyses

Dry matter, crude ash, crude protein, crude fibre, and ether extract contents of the experimental diets were analysed according to standard procedures [33]. Gross energy concentration in diets was analysed using a KL-12Mn adiabatic bomb calorimeter (Precyzja-BIT, Bydgoszcz, Poland).

### 2.3. Analysis of Biochemical Blood Parameters

Glucose concentration and enzyme activities (alanine aminotransferase, alkaline phosphatase, amylase, aspartate aminotransferase, creatine kinase, γ-glutamyltransferase and lipase) in blood plasma were determined spectrophotometrically on a MAXMAT PL biochemical analyser (Erba Diagnostics France SARL, Montpellier, France) using ELITech reagent kits (ELITech Group, Puteaux, France).

### 2.4. Analysis of Prooxidant—Antioxidant Balance in Blood and Breast Muscle

Before the analysis, pectoral muscle samples (0.2 g) were homogenized in 0.8 mL of ice-cold physiological saline and centrifuged (12,850× *g*, 10 min, 4 °C). The obtained supernatants were further treated as blood plasma samples. Based on pilot analyses, an appropriate dilution of samples was determined to fit a linear range of the method. The balance between prooxidants and antioxidants in blood plasma and breast muscle was determined using the spectrophotometric method of Koliakos and Hamidi Alamdari [34], which is based on simultaneous redox and enzymatic reactions, using 3,3′,5,5′-tetramethylbenzidine and its cation. The absorbance was read at 450 nm and 620 nm, as a reference wavelength, using a SpectraMax iD3 microplate reader (Molecular Devices, San Jose, CA, USA). The balance values were calculated from a standard curve prepared using 1 mM H_2_O_2_ as representative of the oxidants, and 6 mM uric acid as representative of the antioxidants, mixed in varying proportions. The results were expressed in arbitrary Hamidi–Koliakos (HK) units per gram of sample or ml of blood plasma.

### 2.5. Measurement of Lipid Peroxidation in Blood and Breast Muscle

Pectoral muscle samples were prepared for the assay as described above. A concentration of thiobarbituric acid-reactive substances (TBARS), a marker of lipid peroxidation, was measured spectrophotometrically using a modified method of Bochicchio et al. [35]. Briefly, 100 μL of blood plasma or muscle supernatant was incubated with 100 μL of 15% trichloroacetic acid (in 0.25 M HCl) and 100 μL of 0.37% thiobarbituric acid (in 0.25 M HCl) for 60 min at 100 °C. After boiling, the samples were cooled to room temperature and centrifuged (10,000× *g*, 10 min). The absorbance of supernatants was measured at 532 nm using a SpectraMax iD3 microplate reader. The TBARS concentration was calculated from a standard curve for malonyldialdehyde.

### 2.6. Myokine Assay

Frozen pectoral muscle samples (0.1 g) were homogenized in 1.5 mL of phosphate-buffered saline, pH 7.4. To disrupt cell membrane and release proteins from cells, homogenates were frozen at −20 °C and stored overnight. Then, the samples were thawed at room temperature and again frozen at −20 °C. After the second thawing, homogenates were centrifuged (12,850× *g*, 10 min, 4 °C) and supernatants were used for analyses.

Concentrations of TNF-α, IL-6 and myostatin in blood plasma and muscle supernatants were determined using ELISA kits (MyBioSource Inc., San Diego, CA, USA) according to the manufacturer’s protocols. Total protein content in supernatants was analysed spectrophotometrically using the Bradford method and Bio-Rad Protein Assay Kit II (Bio-Rad, Hercules, CA, USA) to express myokine concentration per mg protein. All measurements of absorbance were performed on a SpectraMax iD3 microplate reader.

### 2.7. Statistical Analysis

Data were analysed by one-way analysis of variance followed by Tukey’s HSD test. Compliance of the data with the normal distribution was checked by Shapiro–Wilk test, while the homogeneity of variance by Levene’s test. To determine birds’ response to the dietary level of PBC, orthogonal polynomial contrasts were used. All analyses were performed using SPSS statistical package ver. 23.0 (IBM Corp., Armonk, NY, USA). The significance of the effect was set at *p* ≤ 0.05.

## 3. Results

The effect of PBC on growth performance (body weight gain, feed intake and feed conversion ratio) is shown in Table 2. Results indicated that there were no statistically significant differences in feed intake, body weight gain and final body weight. Only feed conversion ratio (FCR) was affected by the experimental factor. The feeding diet supplemented with 100 mg/kg PBC significantly improved FCR as compared to the control group, and there was a linear response of birds. In the case of starter diets, the productive traits were not submitted to statistical analysis due to the group housing of birds.

The activity of alkaline phosphatase in blood was the lowest in birds fed a diet supplemented with 60 mg/kg PBC and similar in the control group and in birds fed a diet supplemented with 100 mg/kg PBC. Alanine aminotransferase and amylase activity were the highest in the control group and the lowest in the group given a diet with 100 mg/kg PBC addition. In contrast, aspartate aminotransferase activity was the lowest in the control group. The highest activity of creatine kinase, γ-glutamyltransferase and lipase was found in the group of chickens fed a diet supplemented with 60 mg/kg PBC. Nonetheless, the differences were not statistically significant. There was also no effect on glucose concentration, which was similar in all groups (Table 3).

The prooxidant–antioxidant balance value was the lowest in the blood of chickens receiving the diet with 60 mg/kg PBC addition, while it was the highest in those fed a diet supplemented with 100 mg/kg PBC. This was in contrast with the balance values obtained for the pectoral muscle. The TBARS concentration in blood was similar in all groups, whereas in the muscle it was the highest in birds fed a diet with 100 mg/kg PBC. However, any difference was not statistically significant (Table 4).

The feeding diet supplemented with PBC affected IL-6 and TNF-α concentrations in blood. IL-6 level was higher in chickens given a diet containing 100 mg/kg PBC than in the control group (*p* < 0.05), while TNF-α concentration was lower in birds given a diet with 60 mg/kg PBC in comparison with the control. The response of IL-6 concentration in blood to dietary level of PBC was linear (*p* < 0.01), whereas that of TNF-α was quadratic (*p* < 0.05). Dietary supplementation with PBC had no effect on myostatin concentration in blood (Table 5).

There was no effect of PBC level on IL-6 and TNF-α concentrations, expressed per mg protein, in pectoral muscle, but MSTN concentration in chickens fed a diet with 60 mg/kg PBC was higher compared to the other groups and there was a quadratic response (*p* < 0.05; Table 5). There was also a highly significant effect of PBC level on TNF-α concentration, expressed per g tissue. Feeding diets supplemented with 60 and 100 mg/kg PBC reduced TNF-α content in pectoral muscle in comparison with the control group and the response was quadratic.

## 4. Discussion

Plants and their extracts may positively affect the performance of broiler chickens. Higher body weight gain has been observed in birds fed diets with the addition of black pepper, turmeric and coriander seeds [36]. It was also found that a hot pepper addition at the dose of 1.5 and 3 g/kg to the diet of Hubbard broiler chicks raised for 45 days resulted in greater body weight gain and increased feed intake as compared to the control and was more effective than oxytetracycline addition [37]. In the present study, body weight gain was unaffected but PBC supplementation improved feed efficiency. The decreased FCR observed in this study is in line with the results of other authors [38,39,40]. The addition of phytobiotics was effective in FCR improvement in broiler chickens, which may be explained by the presence of many bioactive substances that beneficially affect birds` health and stimulate digestive function [37].

Biochemical blood parameters confirmed the good health status of birds as feeding PBC-supplemented diet did not affect liver and pancreatic enzyme levels, which indicated that these organs were not damaged. Previous studies with phytobiotics have demonstrated that these supplements beneficially influence biochemical blood profile by reducing alanine aminotransferase, aspartate aminotransferase and uric acid levels [38,39], by increasing total protein and albumin content and improving lipid profile [40] as well as by enhancement of antioxidant defence [41]. However, in the present study, the prooxidant–antioxidant balance in blood and muscle tissue was not improved. Also, the degree of lipid peroxidation, evaluated based on the TBARS concentration, was not reduced. The lack of antioxidative effect of PBC might result from a low level of dietary supplementation, administration route and duration of supplementation as compared to other studies with phytobiotics. Recently, it has been shown that chickens receiving drinking water with 0.05, 0.1 and 0.25 mL/L phytobiotic containing cinnamon oil (3000 mg/L) and citric acid (150 mg/L) had lower malonyldialdehyde concentration, higher catalase activity and higher ferric reducing ability of plasma in blood in comparison with the control group [41]. The effect of supplementation was more effective when this phytobiotic was administered continuously throughout the whole 42 day-long rearing period than periodically at days 1–7, 15–21 and 29–35 of rearing [41]. Other research, performed on Japanese quails exposed to chronic intermittent cold stress, showed that dietary supplementation with 50 or 100 mg/kg soapwort extract, containing more than 40% triterpenoid saponins, reduced malonyldialdehyde concentration in the liver and heart and increased glutathione peroxidase activity in the liver [21]. The reduction of malonyldialdehyde concentration in blood was also found in quails fed a diet supplemented with 0.4–1.6 g/kg black–red pepper oil mixture, while there was no effect on superoxide dismutase, catalase and glutathione peroxidase [42]. Lower TBARS concentration in fresh and stored meat was also found in broiler chickens fed for 42 days on a diet supplemented with 20 g/kg herbal mixtures composed of onion (70%), thyme (25%), mint (5%) or onion (30%), garlic (20%), oregano (25%), fennel (10%), mint (5%), turmeric (5%) and ginger (5%) [43]. These results indicate the need for further research in which total antioxidant capacity as well as superoxide dismutase, catalase and glutathione peroxidase activities are measured in blood, muscle and other organs of broiler chickens. Also, other factors such as administration route or duration of supplementation should be taken into consideration to elucidate the effect of the PBC used in the present study. In our research, the feeding of experimental diets was continued for 35 days and probably a longer period (42 days) could reveal the antioxidative potential of the additive, which was used at very low dietary levels (60 and 100 mg/kg).

Several muscle-specific interleukins have been shown to play an important role in normal satellite cells and myoblast proliferation, differentiation and muscle remodeling in response to different type of factors. Modulation of interleukin levels, genes and miRNA related to these interleukins using dietary factors seems to be not only a promising option for the treatment of skeletal muscle diseases (myopathies) but also a tool for improving selected production parameters of broiler chickens and ultimately improving the quality of meat.

TNF-α is a cytokine involved in several processes in muscle tissue [44]. This proinflammatory cytokine has been shown to have a physiological role in muscle repair [45] and myogenesis [46]. TNF-α levels are increased during cachectic muscle wasting (exerted through inhibition of myogenic differentiation and enhanced apoptosis) and chronic muscle degeneration, inflammatory myopathies and regeneration processes which are linked to primary muscle disorders. It was shown that curcumin reduced the level of TNF-α in various tissues, and also reduced some disease symptoms associated with the inflammatory process in vitro and in vivo [47]. McFarlin et al. [48] showed curcumin supplementation reduced creatine kinase and TNF-α during recovery but had no effect on subjective muscle soreness. The use of curcumin reduced the subjective perception of the intensity of muscle pain, reduced muscle damage through the decrease in creatine kinase, increased muscle performance and had an anti-inflammatory effect by modulating the proinflammatory cytokines, such as TNF-α, IL-6 and IL-8, and might have a slight antioxidant effect [49]. Similar effects were observed in the cases of terpenes [50] and capsaicin [51] that reduced the level of TNF-α. Other compounds present in the PBC used in the current experiment, such as saponins, have previously been described in relation to inhibition of TNF-α or decreasing its level in various tissues in human and animal models [52,53]. All these findings indicate that the reduction or even inhibition of TNF-α in various tissues, including muscle tissue and blood, is an extremely beneficial phenomenon, not only due to the reduction of inflammatory processes but also due to a beneficial effect on the processes of tissue regeneration and protection of muscle.

In our study, the level of TNF-α in blood was reduced in chickens fed diet supplemented with 60 mg/kg PBC as compared to the control, while in muscle tissue its concentration was decreased by both dietary PBC levels. This means that this unique dietary supplement may affect this cytokine even at the lower dose. Moreover, the results suggest that muscle tissue may be a target for this feed additive, despite the lack of effect on oxidative stress markers, i.e., TBARS and prooxidant–antioxidant balance.

IL-6 is another major regulator of myogenesis that is secreted by infiltrating macrophages and neutrophils, fibro/adipogenic progenitors and muscle itself [54,55]. It has previously been described as the cytokine responsible for satellite cell proliferation in vitro [56]. In the case of broilers, most of the research related to IL-6 was mainly related to inflammatory processes in the digestive system, oxidative stress and heat stress [57]. In a model of IL-6-deficient mice subjected to hind limb muscle overload (denervation of one limb muscle and function compensation of the other muscle), the phenomenon of compensatory muscle hypertrophy occurred [56]. Interestingly, it was noted that in hypertrophic muscles, the production of IL-6 increased 40-fold, and muscles lacking IL-6 grew 20% less. It was further established that IL-6 is necessary for the proliferation of satellite cells and the fusion of their nuclei with muscle fibres [56]. It was also found that IL-6 is associated with the proliferation of satellite cells during regeneration of muscle tissue [55]. All these studies clearly show that the presence of IL-6 and its corresponding high level is necessary for a number of processes that are needed for the protection of various tissues, their regeneration and their proper development. Therefore, it can be supposed that the higher level of IL-6 in the blood of chickens fed PBC-supplemented diets may be related to an improvement in the quality of muscle tissue and its protection against pathological processes that accompany the intensive rearing of poultry.

MSTN is a member of the transforming growth factor-β family expressed predominantly in the muscle tissues. It inhibits muscular development through cellular differentiation of developing somites during the embryonic stage and growth of myofibrillar cells during the adult stage in animals, and is mostly known as a typical negative regulator of myogenesis [58,59]. The MSTN gene expression and protein level may be modulated by nutrients, which has been shown for berberine [60]. Rapid improvement of muscling in meat producing animals through lowering the gene expression of MSTN, its protein and receptors, may be one of the most important approaches in the livestock and poultry industry. So far, there have been no studies in regard to this myokine in broiler chickens. In the present study, it was found that MSTN concentration in pectoral muscle was increased by feeding a diet supplemented with 60 mg/kg PBC but only when it was expressed per mg protein. These results clearly indicate that MSTN content in muscle is highly dependent on the protein content in muscle tissue. In the breast muscle of chickens, the protein content (mg/g tissue) was 44.13, 37.52, and 44.28 for the control, 60 mg/kg PBC and 100 mg/kg PBC group, respectively. There was no significant effect of PBC level on protein content but it was the lowest in the group fed a diet with 60 mg/kg PBC that also showed the highest MSTN content. Therefore, it can be stated that MSTN content is inversely proportional to protein content in the muscle. Nonetheless, the results are difficult to explain and it remains to be elucidated which bioactive compounds of the PBC are responsible for the effect on MSTN and why there was no linear response of birds.

**Figure 1:**
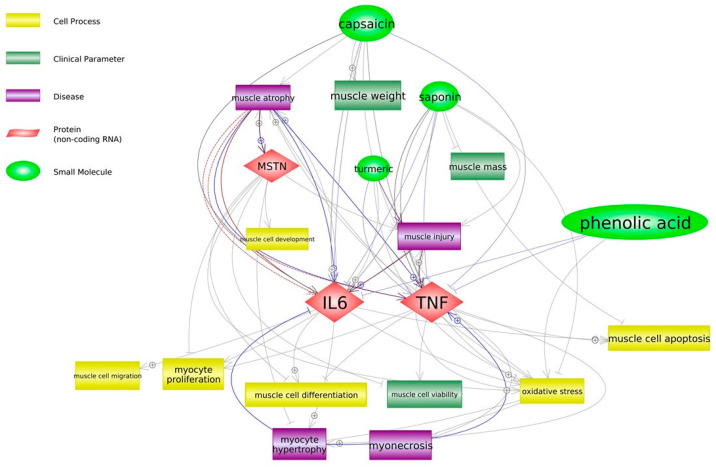
Potential effect of bioactive ingredients of phytobiotic composition on muscle tissue in broiler chickens (Pathway Studio^®^ Mammal Plus, Elsevier).

Our study presented new findings on the mechanisms of action of PBC containing red pepper (source of capsaicin), white mustard (glucosinolates), soapwort (saponins), calamus (phenols) and turmeric (curcumin) on broiler chickens. Through their effect on myokines, these bioactive compounds may be involved in muscle tissue injury (inflammation) regeneration and the accompanying processes such as cell activation, proliferation, migration and differentiation. The scheme of their potential activity in relation to our findings is proposed in Figure 1.

All these processes are related to normal muscle physiology and general muscle tissue health, which is crucial for high quality meat obtained from broiler chickens. The current study gives a new perspective on certain issues that have not been previously analysed in the broiler chicken model, which makes the results difficult to interpret. Moreover, based on the obtained data, it is evident that there is a need to check other interleukins, possibly also those that have not been tested in poultry so far.

## 5. Conclusions

It can be concluded that dietary supplementation with 60 mg/kg PBC containing red pepper fruit, white mustard seed, soapwort root and calamus rhizome neither affected the growth performance nor the basic biochemical blood parameters of broiler chickens, including lipid peroxidation products and prooxidant–antioxidant balance. However, it contributed to greater IL-6 and lower TNF-α concentration in blood, increased myostatin content and reduced that of TNF-α in pectoral muscle.

The results also showed that dietary supplementation with phytobiotic composition at the level of 100 mg/kg diet improved feed efficiency in broiler chickens and may improve quality and economy of broiler meat production. Of particular importance are the decrease in TNF-α concentration in pectoral muscle and an increase in IL-6 content in blood. These changes beneficially influence tissue regeneration and protection of pectoral muscle against pathophysiological processes that may occur during intensive rearing of broiler chickens.

## Figures and Tables

**Table 1:** Ingredient and chemical composition of the PBC-unsupplemented and PBC-supplemented diets.

Item	Diets		
	Starter (1–10 Day)	Grower (11–28 Day)	Finisher (29–35 Day)
Ingredients (%)			
Corn meal	32.44	32.07	31.80
Soybean meal	30.70	30.11	24.68
Wheat	30.00	30.00	35.00
Rapeseed oil	3.08	4.53	5.41
Premix with salinomycin	3.00	3.00	3.00
Monocalcium phosphate	0.41	0.24	0.11
Methionine	0.28	0.03	
Lysine HCl	0.07		
Calcium carbonate	0.02	0.02	
Nutrient content (%)			
Dry matter	90.68 ^1^/91.17 ^2^	90.87 ^1^/90.90 ^2^	90.81 ^1^/90.98 ^2^
Crude protein	20.25 ^1^/20.44 ^2^	19.75 ^1^/19.25 ^2^	17.56 ^1^/17.88 ^2^
Crude ash	4.40 ^1^/4.79 ^2^	4.20 ^1^/4.21 ^2^	3.74 ^1^/3.62 ^2^
Crude fat	5.41 ^1^/5.92 ^2^	6.76 ^1^/6.98 ^2^	8.22 ^1^/8.02 ^2^
Crude fibre	2.54 ^1^/2.48 ^2^	2.67 ^1^/2.62 ^2^	2.76 ^1^/2.69 ^2^
Gross energy (MJ/kg)	17.6 ^1^/18.2 ^2^	18.2 ^1^/18.9 ^2^	18.6 ^1^/18.8 ^2^

^1^ Control diets. ^2^ Diets supplemented with 60 or 100 mg/kg of phytobiotic composition.

**Table 2:** Growth performance parameters in chickens fed diets supplemented with 60 and 100 mg/kg PBC.

Parameter	PBC (mg/kg)			SEM	*p* Value	Contrast
	0	60	100			
	**Starter (1–10 day of life)**					
Feed intake, g	272.1	288.2	272.8			
Body weight gain, g	111.9	118.9	107.6			
FCR	1.36	1.34	1.41			
	**Grower + finisher (11–35 day of life)**					
Feed intake, g	2731	2736	2538	87.2	0.192	
Body weight gain, g	2080	2166	2098	58.4	0.563	
FCR	1.39 ^b^	1.37 ^a,b^	1.22 ^a^	0.049	0.032	linear
Final body weight, g	2343	2444	2388	58.3	0.479	

^a,b^ means followed by different letters within the same line are statistically different (*p* < 0.05).

**Table 3:** Biochemical blood indices in chickens fed diets supplemented with 60 and 100 mg/kg PBC.

Parameter	PBC (mg/kg)			SEM	*p* Value
	0	60	100		
ALP ^1^ (U/L)	4064	3446	4081	305.6	0.643
ALT ^2^ (U/L)	23.7	20.4	17.7	1.50	0.272
Amylase (U/L)	549	454	398	44.8	0.390
AST ^3^ (U/L)	356	377	405	22.3	0.682
CK ^4^ (U/L)	30,125	37,555	35,548	3665.0	0.708
GGTP ^5^ (U/L)	17.9	20.9	18.6	0.63	0.124
Glucose (mmol/L)	16.2	16.7	16.5	0.16	0.501
Lipase (U/L)	6.37	7.07	6.19	0.558	0.804

^1^ Alkaline phosphatase. ^2^ Alanine aminotransferase. ^3^ Aspartate aminotransferase. ^4^ Creatine kinase. ^5^ γ-glutamyltransferase.

**Table 4:** Oxidative stress markers in chickens fed diets supplemented with 60 and 100 mg/kg PBC.

Parameter	PBC (mg/kg)			SEM	*p* Value
	0	60	100		
Blood					
PAB ^1^ (HKU/mL)	955	821	1141	69.8	0.172
TBARS ^2^ (μmol/L)	0.32	0.36	0.37	0.038	0.854
Pectoral muscle					
PAB (HKU/g)	7434	7866	7095	216.3	0.354
TBARS (μmol/g)	2.43	1.99	3.47	0.483	0.444

^1^ Prooxidant–antioxidant balance. ^2^ Thiobarbituric acid-reactive substances.

**Table 5:** Myokine concentrations in blood and pectoral muscle of broiler chickens fed diets supplemented with 60 and 100 mg/kg PBC.

Parameter	PBC (mg/kg)			SEM	*p* Value	Contrast
	0	60	100			
Blood						
IL-6 ^1^(pg/mL)	404 ^a^	580 ^a,b^	605 ^b^	33.6	0.022	linear
TNF-α ^2^ (ng/L)	10.87 ^b^	9.00 ^a^	9.98 ^a,b^	0.313	0.045	quadratic
MSTN ^3^ (ng/mL)	0.48	0.54	0.25	0.108	0.533	
Pectoral muscle						
IL-6 (ng/g tissue)	19.5	14.6	20.2	2.04	0.492	
IL-6 (pg/mg protein)	520	536	471	72.7	0.934	
TNF-α (pg/g tissue)	148 ^b^	112 ^a^	107 ^a^	4.19	<0.001	quadratic
TNF-α (pg/mg protein)	3.85	3.52	2.68	0.267	0.190	
MSTN (ng/g tissue)	78	110	83	6.7	0.111	
MSTN (ng/mg protein)	2.01 ^a^	3.46 ^b^	1.93 ^a^	0.258	0.019	quadratic

^1^ Interleukin-6. ^2^ Tumor necrosis factor-α. ^3^ Myostatin. ^a,b^ Mean values in the rows with different superscript letters differ significantly (*p* ≤ 0.05).

## Data Availability

The data presented in this study are available on request from the corresponding author.
